# Hereditary factors in basal cell carcinoma of the skin: a population-based cohort study in twins—reply

**DOI:** 10.1054/bjoc.1999.0953

**Published:** 1999-12-08


1. The first limit to inference is the power of the study, which is insufficient to rule out a genetic effect. Although the CE model was reported as fitting the data best, the confidence intervals for A (additive genetic effects) in the AE and ACE models are very large. Both confidence intervals for A and C (shared environment) contains 0 for the ACE model. A genetic effect as large as 60% cannot be ruled out by this study. Accepting C in place of A for discontinuous traits is logistically difficult and would require a sample size of at least 20 000 twin pairs given the prevalence of basal cell carcinomas of around 2% (Neale et al, 1994).
2. The authors do not explore or comment on the significant common environmental influence that has been observed, which could merely be due to the age-dependence of BCCs. The mean age of onset for sporadic BCC is around 65 years of age, whilst familial tumours usually occur at younger age and are often multiple (Kimonis et al, 1997). Failure to account for age may mask the importance of a heritable component in the data.
3. No account was taken of body sites. In genetically susceptible families, it is well recognized that tumours are more often found on the trunk than the face (Kimonis et al, 1997). By combining all body sites in the analysis, it may have masked a site-specific genetic effect.
4. Loss of heterozygosity studies have shown that 60% of BCCs show loss of chromosome 9q, which harbour the patched (PTC) gene (Gailani et al, 1992). Germline mutations in the PTC gene are found in patients with naevoid BCC syndrome, a family cancer syndrome characterized by multiple early onset BCCs and developmental defects (Johnson et al, 1991). A genetic basis for this disease is therefore likely and can only be adequately discounted in much larger studies using designs that take into account the known biological properties of the disease.

*V Bataille[1,2], H Snieder[2], A MacGregor[2] and T Spector[2]*
*[1]Academic Department of Dermatology, St Bartholomew's and Royal London School of Medicine and Dentistry, London E1, UK; [2]Twin Research and Genetic Epidemiology Unit, St Thomas Hospital, London SE1, UK*

### REFERENCES

Gailani MR, Bale SJ, Leffell DJ et al (1992) Developmental defects in Gorlin's syndrome related to putative tumour suppressor gene on chromosome 9. *Cell* **69**: 111–117

Johnson RL, Rothman AL, Xie J et al (1996) Human homolog of patched, a candidate gene for the basal cell nevus syndrome. *Science* **14**: 1668–1671

Kimonis VE, Goldstein AM, Pastakia B et al (1997) Clinical manifestations in 105 persons with the nevoid basal cell carcinoma syndrome. *Am J Med Genet* **31**: 299–308

Neale MC, Eaves LJ and Kendler KS (1994) The power of the classical twin study to resolve variation in threshold traits. *Behavior Genet* **24**: 239–258

### Hereditary factors in basal cell carcinoma of the skin: a population-based cohort study in twins—reply

**Sir,**

We thank Dr Bataille and her colleagues for their interest in our paper on hereditary factors in basal cell carcinoma of the skin (BCC), based on large population-based sample of adult twins from Finland (Milan et al, 1998). They write that we concluded that genetic factors are not necessary to explain the distribution of BCC in twins, and raise a number of issues that they believe we should have addressed.

It should first be pointed out that our conclusion was (as stated in the last sentence of the abstract) that the results confirm the major role of environmental factors, which was based on our results from various genetic models shown in Table 4. In the Introduction, we state that genetic disorders are known to be associated with the development of BCC; in the Discussion, we suggest that these disorders do not appear to be of major importance at the population level.

The decision to emphasise the best-fitting model, CE, with shared (C) and unshared (E) environmental effects, derives from the logic of model-fitting, which is to seek a model which accounts for the observed data in the most parsimonious fashion, as advocated by the standard text on twin analyses (Neale and Cardon, 1992). Such a model is more easily falsifiable in a subsequent study than a more complex model, and we look forward to other analyses of BCC from large, population-based twin or family data sets. The more complex ACE-model did indeed contain the point estimate of zero for both additive genetic effects (A) and shared environmental effects (C), but the pure environmental model, E-model, could be rejected. Nonetheless, in the ACE model, the point estimate for the additive genetic component was 7.7%, leaving over 90% of the inter-individual variability in the liability to BCC to be attributed to environmental effects. The remaining AE model, which Dr Bataille appears to be advocating, had a poorer fit than the CE or ACE models.

Precisely because of the power issue (only four MZ and seven DZ concordant pairs), we could not account for age effects in genetic modelling. It certainly would be desirable to have more twin pairs for such an analysis, but most twin study samples in the world are considerably smaller than ours.

The mean age of diagnosis of the twins in the concordant pairs was 64.1 years (range 38–82 years, both extremes being MZ male twins, four out of 22 twins being diagnosed prior to age 60 years). The twins from concordant pairs were not markedly younger than were the other BCC patients on average. Failure to account for age in genetic modelling thus appears to be an unlikely explanation for not observing genetic effects.

Three-quarters (73%) of all BCCs registered with the Finnish Cancer Registry between 1953 and 1995 were located in the head and neck (unpublished data), compared with 68% of the twins from the concordant pairs of the present study. One MZ pair (ages at diagnosis 38 years and 43 years) and one DZ pair (60 and 61 years) were concordant for having a trunk location.

The identification of the role of the patched gene in the pathogenesis of BCC is a very important observation, which we also indicated (with three references) in our Discussion. However, the basal cell naevus syndrome is a rare disease, and accounts for only a very minor fraction of all BCCs in the population. The role of germline mutations in the patched gene in sporadic BCC cases should be assessed by the careful study of an unselected BCC

 *© 2000 Cancer Research Campaign*

patient population and appropriate controls. A relatively small number of patients should suffice to indicate whether such mutations are relevant in sporadic BCC.

*T Milan, J Kaprio, PK Verkasalo, CT Jansen, L Teppo and M Koskenvuo, Department of Public Health, University of Turku, Lemminkäisenkatu 1, 20520 Turku, Finland*

**REFERENCES**

Milan T, Kaprio J, Verkasalo PK, Jansén CT, Teppo L, Koskenvuo M (1998) Hereditary factors in basal cell carcinoma of the skin: a population-based cohort study in twins. *Br J Cancer* 1998; **78**: 1516–1520.
Neale MC and Cardon LR (1992) *Methodology for Genetic Studies of Twins and Families*. Kluwer Academic, Dordrecht

### The relation of gelatinase (MMP-2 and -9) expression with distant site metastasis and tumour aggressiveness in colorectal cancer

**Sir,**

We read with great interest the report by Parsons et al (1998) on matrix metalloproteinase (MMP)-2 and -9 expression in gastrointestinal malignancy.

We agree with the authors on the significant role of MMP-2 and -9 in the transformation of a tumour from the benign to the malignant state by enabling the tumour cells to infiltrate blood vessels and lymphatics allowing metastasis to a distant site. However, since the distant site metastasis is an important aspect of the malignancy, it brings a question about the specimens taken from the patients having colorectal cancer, whether they do have metastasis or not. The metastatic colorectal cancers should have been distinguished from the ones that have not metastasized yet. After then it would be much more meaningful to make a comparison between metastatic and non-metastatic groups according to their MMP-2 and -9 expressions in the primary sites.

Additionally, it would be also interesting to find out corresponding results when one considers that some cases are likely to have different organ preferences of metastasis. Moreover, as the MMPs have been implicated in tumour progression (Liotta and Stetler-Stevenson, 1990), with recent evidence suggesting that MMPs are key regulators of the growth of tumours at both primary and metastatic sites (Chambers and Matrisian, 1997), it would be more meaningful to investigate the expressions of MMP-2 and -9 at metastatic sites as well as at primary sites.

On the other hand, all colorectal cancers were classified by the Dukes' staging system as well as other measures. Since only Dukes' stage A, B and C patients were defined in the results, it is highly probable that the original Dukes' staging system (Dukes, 1932) was used in the subdivision of colorectal cancers. Tumour stage 'D' was not included in the original Dukes' staging system and also is not routinely included in Astler–Coller classification (Astler and Coller, 1954) of carcinoma of the colon and rectum which represents modification of classification proposed by Dukes. However, it has become commonly used to represent distant metastasis after addition into Astler–Coller classification by Turnbull et al (1967).

Since stage 'D' is highly related to our topic and was not considered in the study we are not convinced of the conclusion that there is no statistically significant correlation between gelatinase expression and any of the recognized measures of tumour aggressiveness.

Consequently, in the article neither there was a subdivision of colorectal cancers as metastatic and non-metastatic nor tumour stage 'D', which represents distant site metastasis has been included in the staging of colorectal cancers. If the parts of the investigation concerning the relation of gelatinase expression with distant site metastasis and tumour aggressiveness were based upon those above mentioned mainstays much more satisfactory results of the study would be possible to be obtained.

*I. H. Güllü, M. Kurdoğlu, Í. Akalın*
*Institute of Oncology, Hacettepe University, Ankara, Turkey*

**REFERENCES**

Astler VB and Coller FA (1954) The prognostic significance of direct extension of carcinoma of the colon and rectum. *Ann Surg* **139**: 846
Chambers AF and Matrisian LM (1997) Changing views of the role of matrix metalloproteinases in metastasis. *J Natl Cancer Inst* **89**: 1260–1270
Dukes CE (1932) The classification of cancer of the rectum. *J Pathol & Bact* **35**: 323
Liotta LA and Stetler-Stevenson WG (1990) Metalloproteinases and cancer invasion. *Semin Cancer Biol* **1**: 99–106
Parsons SL, Watson S, Collins H, Griffin N, Clarke P and Steele R (1998) Gelatinase (MMP-2 and -9) expression in gastrointestinal malignancy. *Br J Cancer* **78**: 1495–1502
Turnbull RB Jr, Kyle K, Watson FR, et al (1967) Cancer of the colon: the influence of the no-touch isolation technic on survival rates. *Ann Surg* **166**: 420–427

© 2000 Cancer Research Campaign

## References

[PDF_00189] ASTLER V. B., COLLER F. A. (1954). The prognostic significance of direct extension of carcinoma of the colon and rectum.. Ann Surg.

[PDF_00191] Chambers A. F., Matrisian L. M. (1997). Changing views of the role of matrix metalloproteinases in metastasis.. J Natl Cancer Inst.

[PDF_00048] Gailani M. R., Bale S. J., Leffell D. J., DiGiovanna J. J., Peck G. L., Poliak S., Drum M. A., Pastakia B., McBride O. W., Kase R. (1992). Developmental defects in Gorlin syndrome related to a putative tumor suppressor gene on chromosome 9.. Cell.

[PDF_00051] Johnson R. L., Rothman A. L., Xie J., Goodrich L. V., Bare J. W., Bonifas J. M., Quinn A. G., Myers R. M., Cox D. R., Epstein E. H. (1996). Human homolog of patched, a candidate gene for the basal cell nevus syndrome.. Science.

[PDF_00053] Kimonis V. E., Goldstein A. M., Pastakia B., Yang M. L., Kase R., DiGiovanna J. J., Bale A. E., Bale S. J. (1997). Clinical manifestations in 105 persons with nevoid basal cell carcinoma syndrome.. Am J Med Genet.

[PDF_00195] Liotta L. A., Stetler-Stevenson W. G. (1990). Metalloproteinases and cancer invasion.. Semin Cancer Biol.

[PDF_00125] Milán T., Kaprio J., Verkasalo P. K., Jansén C. T., Teppo L., Koskenvuo M. (1998). Hereditary factors in basal cell carcinoma of the skin: a population-based cohort study in twins.. Br J Cancer.

[PDF_00056] Neale M. C., Eaves L. J., Kendler K. S. (1994). The power of the classical twin study to resolve variation in threshold traits.. Behav Genet.

[PDF_00197] Parsons S. L., Watson S. A., Collins H. M., Griffin N. R., Clarke P. A., Steele R. J. (1998). Gelatinase (MMP-2 and -9) expression in gastrointestinal malignancy.. Br J Cancer.

[PDF_00200] Turnbull R. B., Kyle K., Watson F. R., Spratt J. (1967). Cancer of the colon: the influence of the no-touch isolation technic on survival rates.. Ann Surg.

